# Supplementary material for: Comparative efficacy and acceptability of non-pharmacological interventions for depression among people living with HIV: A protocol for a systematic review and network meta-analysis
Source: PLoS One. 2023 Jun 27;18(6):e0287445. doi: 10.1371/journal.pone.0287445 (PMC10298765; doi:10.1371/journal.pone.0287445)
Supplement: S1 Appendix — (DOCX) [file pone.0287445.s001.docx]

# S1 Appendix. Search String

Take PubMed as an example, the following keywords and Medical Subject Headings (MESH) will be used for searching articles:

#1 "HIV"[Mesh] OR "Acquired Immunodeficiency Syndrome"[Mesh] OR "HIV Infections"[Mesh]

#2 "Depression"[Mesh] OR "Depressive Disorder"[Mesh]

#3 (Human Immunodeficiency Virus*[Title/Abstract] OR Immunodeficiency Virus*, Human[Title/Abstract] OR Virus*, Human Immunodeficiency[Title/Abstract] OR Human T Cell Lymphotropic Virus Type III[Title/Abstract] OR Human T-Cell Lymphotropic Virus Type III[Title/Abstract] OR Human T-Cell Leukemia Virus Type III[Title/Abstract] OR Human T Cell Leukemia Virus Type III[Title/Abstract] OR LAV-HTLV-III[Title/Abstract] OR Lymphadenopathy-Associated Virus*[Title/Abstract] OR Lymphadenopathy Associated Virus[Title/Abstract] OR Virus*, Lymphadenopathy-Associated[Title/Abstract] OR Human T Lymphotropic Virus Type III[Title/Abstract] OR Human T-Lymphotropic Virus Type III[Title/Abstract] OR AIDS Virus*[Title/Abstract] OR Virus*, AIDS[Title/Abstract] OR AIDS[Title/Abstract] OR Acquired Immune Deficiency Syndrome Virus[Title/Abstract] OR HTLV-III[Title/Abstract] OR HIV Infection[Title/Abstract] OR Infection*, HIV[Title/Abstract] OR HTLV-III-LAV Infection*[Title/Abstract] OR Infection*, HTLV-III-LAV[Title/Abstract] OR T-Lymphotropic Virus Type III Infections, Human[Title/Abstract] OR T Lymphotropic Virus Type III Infections, Human[Title/Abstract] OR HTLV-III Infection*[Title/Abstract] OR HTLV III Infections[Title/Abstract] OR Infection*, HTLV-III[Title/Abstract] OR HIV Coinfection*[Title/Abstract] OR Coinfection*, HIV[Title/Abstract])

#4 (Depression*[Title/Abstract] OR Depressive Symptom*[Title/Abstract] OR Symptom*, Depressive[Title/Abstract] OR Emotional Depression*[Title/Abstract] OR Depression*, Emotional[Title/Abstract] OR Depressive Disorders[Title/Abstract] OR Disorder*, Depressive[Title/Abstract] OR Neuros*, Depressive[Title/Abstract] OR Depressive Neuros*[Title/Abstract] OR Depression*, Endogenous[Title/Abstract] OR Endogenous Depression*[Title/Abstract] OR Depressive Syndrome*[Title/Abstract] OR Syndrome*, Depressive[Title/Abstract] OR Depression*, Neurotic[Title/Abstract] OR Neurotic Depression*[Title/Abstract] OR Melancholia*[Title/Abstract] OR Unipolar Depression*[Title/Abstract] OR Depression*, Unipolar[Title/Abstract])

#5 (#1 OR #3) AND (#2 OR #4)

#6 ("Clinical Trials as Topic"[MeSH] OR randomized controlled trial[Publication Type] OR controlled clinical trial[Publication Type] OR randomized[Title/Abstract] OR placebo[Title/Abstract] OR randomly[Title/Abstract] OR trial[Title])

#7 ("Animals"[MeSH] NOT "Humans"[MeSH])

#8 #6 NOT #7

#9 #5 AND #8
