# Supplementary material for: Comparative efficacy and acceptability of non-pharmacological interventions for depression among people living with HIV: A protocol for a systematic review and network meta-analysis
Source: PLoS One. 2023 Jun 27;18(6):e0287445. doi: 10.1371/journal.pone.0287445 (PMC10298765; doi:10.1371/journal.pone.0287445)
Supplement: Attachment — Submitted filename: PLOS Review.docx [file pone.0287445.s003.docx]

Overall, this is an intriguing idea and I think a very worthy addition to the literature. However, some careful editing is needed.

**Abstract**

“We will employ a network meta-analysis to synthesize all available evidence for each outcome and obtain a comprehensive ranking of all interventions for the global network and for low-income and middle-income countries network only.” I’m not sure what network is being referenced. Also, should it be countries’ network?

**Introduction**

Paragraph 2, sentence 3 “Furthermore, it may cause potential somatic distress,

drug interactions with HIV medications, drug abuse, and lethality in overdose[13].”

The support is poor as it is from the introduction to a 2009 paper and not primary literature. The above sentence in the paper needs better citation given pharmacotherapy is standard of care in the general population in high income countries and generally safe. I’m not tracking with how antidepressants are causing drug abuse. Also, most SSRIs are hard to overdose on.

Paragraph 3- low cost- varies widely. Depends on who is giving it. You go on to explain why it is not low cost- requirement of trained staff and the limited access to such staff.

Paragraph 4- You are implying these non-pharmacologic interventions bring benefits. I think you are actually weighing *if* they bring benefit and how much

Grammatical errors- ex. Like 87-88 should be “have been provided.” Not “has been provided”.

Network meta-analysis- should have a citation.

**Types of Participants.**

“For the clinical difference, we will exclude RCTs in which 20% or more of the participants are suffering from bipolar or psychotic depression, but not involving patients with other comorbid psychiatric disorders (eg. anxiety disorder).” I am not sure what this is saying. Please re-word the sentence.

“We will also exclude studies in which participants with a serious concomitant medical illness. “ HIV is a serious medical illness. I think you mean other than HIV but the sentence should be reworded.

**Types of Intervention**

Synthetic comparison set- needs a definition

Table 1 has inconsistent capitalization.
