# Supplementary material for: Comparative efficacy and acceptability of non-pharmacological interventions for depression among people living with HIV: A protocol for a systematic review and network meta-analysis
Source: PLoS One. 2023 Jun 27;18(6):e0287445. doi: 10.1371/journal.pone.0287445 (PMC10298765; doi:10.1371/journal.pone.0287445)
Supplement: Attachment — Submitted filename: Response to Reviewers.docx [file pone.0287445.s005.docx]

Dear editors and reviewers,

Thank you very much for your constructive comments and suggestions on our manuscript entitled “Comparative efficacy and acceptability of non-pharmacological interventions for depression among people living with HIV: A protocol for a systematic review and network meta-analysis” (PONE-D-22-17925). We appreciate the time and effort that you and the reviewers dedicated to providing feedback on our manuscript. We have carefully considered the comments and revised our manuscript accordingly. Changes to our previous manuscript are marked in red on the revised manuscript. The detailed point-by-point responses to the reviewer’s comments and concerns are listed below:

Responses to Journal Requirements:

Comment 1: Please review your reference list to ensure that it is complete and correct. If you have cited papers that have been retracted, please include the rationale for doing so in the manuscript text, or remove these references and replace them with relevant current references. Any changes to the reference list should be mentioned in the rebuttal letter that accompanies your revised manuscript. If you need to cite a retracted article, indicate the article’s retracted status in the References list and also include a citation and full reference for the retraction notice.

Response 1: We sincerely appreciate the valuable comments. We have ensured that our reference list is complete and correct. We have not cited papers that have been retracted. Furthermore, we have updated reference 1 (World Health Organization). The changes to our reference list are marked in red on our revised manuscript.

Special thanks to you for your valuable comments.

Responses to the reviewers’ comments:

Reviewer #1:

Comment 1: I suggest an additional sentence to clarify the reason for exclusion of other types of depression being that you think these will interact with the estimated effects (?), which is not the case for co-morbid psychiatric disorders. In effect, you are conditioning by design, although that won't be clear to everyone.

Response 1: We sincerely appreciate the valuable comments. The revised texts read as follows:” To reduce clinical heterogeneity, we will exclude RCTs involving 20% or more participants with bipolar depression, treatment-resistant depression, psychotic depression, or peripartum depression, but not studies involving participants with other comorbid psychiatric disorders (e.g., anxiety disorder).” (Pages 6 to 7, lines 118 to 121)

Comment 2: I would keep an open mind to the possibility of splitting nodes according to levels of dose, because you may find adequate data with which to do this (more commonly we don't). As you have it written, defining multiple nodes for different doses of the same intervention would be a protocol deviation, which would be unfortunate. I suggest "We will consider different intensity levels or subtypes within the same type of non-pharmacological intervention as the same node in the network analysis in the first instance, or as different nodes if adequate data are available.”

Response 2: Thanks for your valuable comments. The revised texts read as follows:“We will consider different intensity levels or subtypes within the same type of non-pharmacological intervention as the same node in the network analysis in the first instance, or as different nodes if adequate data are available.” (Page 7, lines 128 to 131)

Comment 3: Okay, your reasons for using SMD are considered and reasonable. In that case, please bear in mind that the estimate of the heterogeneity variance will be critical to accurate interpretation of the results. You are assuming this parameter to be common across comparisons - that's reasonable and often the only option given the amount of data. That being so, if this estimate is not precise, it will be difficult to interpret the SMDs for the relative effects.

Response 3: Thank you for this valuable comment. Considering the importance of heterogeneity, we have added details in this regard. The revised texts read as follows:

“We will assume a common heterogeneity variance parameter (τ) for each network. We will compare the posterior distribution of the estimated heterogeneity with its empirical distributions to account for the magnitude of the estimated heterogeneity[1]. Specifically, we will use the first and third quantiles of their predictive distributions to characterize the amount of heterogeneity as low, moderate, or high[2].” (Page 14, lines 269 to 274)

“If we find significant heterogeneity and/or inconsistency, we will conduct subgroup analyses or network meta-regression analyses to explore the possible sources.” (Page 15, lines 302 to 303)

Comment 4: Thanks for making those updates regarding heterogeneity estimation and exploration. Strictly, I^2 is not a test, so please drop that word.

Response 4: We sincerely thank you for your careful reading. The revised texts read as follows:” Heterogeneity will be evaluated using Cochran’s Q test and *I^2^*.” (Page 12, line 244)

Comment 5: You might also note that you can compare the fit of the two models as an additional 'global' index of incoherence (although null difference is not evidence of coherence).

Response 5: Thanks for your valuable comments. We will calculate the posterior mean of residual deviance to access model fit. The closer the posterior mean of residual deviance is to the number of data points, the better the model fit[3]. (Page 13, lines 256 to 258)

Comment 6: I urge you strongly not to compare results of models with fixed or random effects terms for the treatment effects. Whereas with coherence we often do not know, in the case of whether treatment effects follow a distribution (random setting) or vary only due to sampling error (fixed setting), it is rare this is not known. Please choose an approach and stick with it, because the choice of term influences the ability to assess coherence. For example, in a network where there are few studies per comparison, and the model has a random effects term for treatment effects, there may be a false conclusion of no loop incoherence simply because the CIs are enlarged (and thus overlap for the indirect and direct estimates). Whilst, in this hypothetical scenario, the random effects term may be appropriate, there is the noted drawback of lesser ability to evaluate coherence. You won't have major problems, because you're using multiple means to evaluate coherence, but the point still stands that the treatment effects term needs to be chosen based on prior knowledge to avoid unwarranted model flexibility.

Response 6: We sincerely appreciate the valuable comments. The revised texts read as follows:” We will use the random-effects model in the Bayesian analyses.” (Page 13, lines 255 to 256)

Comment 7: Please bear in mind the potential for results to vary between the Bayesian and frequentist settings. I suggest you also estimate treatment effects with mvmeta, compare them to those from OpenBUGS, and note the differences before looking at coherence metrics.

Response 7: Thanks for your valuable comments. The revised texts read as follows:” All analyses of the primary outcomes will be duplicated within a Frequentist framework using the ‘netmeta’ packages in R (version 4.1.2) software.” (Page 14, lines 274 to 275)

Comment 8: Lastly, please add a statement of where you will disseminate the study findings and data?

Response 8: Many thanks to your kind reminder. The revised texts read as follows:” This study will use secondary data and therefore does not require ethical approval. The results of this study will be disseminated through peer-reviewed publication.” (Page 3, lines 49 to 51)

Special thanks to you for your valuable comments.

Reviewer #3:

Comment 1: it would be grateful if they included a sentence in the methods on how to deal with missing data.

Response 1: Thanks for your valuable comments. When standard deviations (SDs) are not reported, we will first use the standard errors (SEs), t-statistics, p values, and so on to calculate the missing SDs. We will also contact the authors of the included study up to three times via email to obtain the relevant missing data and seek clarifications, which will be regarded as unobtainable if no clarification is provided within six weeks. (Page 11, lines 201 to 205)

Comment 2: what procedure to consider in the case of incomparable nodes of the interventions examined.

Response 2: Thank you for this valuable comment. We will only consider any non-pharmacological interventions recommended by current guidelines. To reduce inconsistency among trials, we will exclude studies that combined non-pharmacological interventions with specific drugs and studies using specific drugs as the comparator. We will consider different intensity levels or subtypes within the same type of non-pharmacological intervention as the same node in the network analysis in the first instance or as different nodes if adequate data are available. Furthermore, before running the network meta-analysis, we will evaluate the transitivity assumption (a fundamental assumption for the validity of indirect comparisons in network meta-analysis[4]) by comparing the distribution of potential effect modifiers across the different comparisons and evaluating consistency models. Important intransitivity might be manifested in the data as a statistical inconsistency (differences between direct and indirect evidence)[4, 5]. We will evaluate local and global inconsistency in Stata (version 15.1) software using the ‘network’ and ‘mvmeta’ packages. If there is no significant inconsistency, we will use the consistency model; otherwise, an inconsistency model will be used[6], and we will conduct sensitivity analyses for our primary outcomes by excluding local inconsistency spots to test the robustness of the results. (Page 7, lines 124 to 131; Page 14, lines 279 to 282; Page 15, lines 290 to 299; Page 16, lines 318 to 320)

Comment 3: I also believe that the overall quality of evidence for each treatment comparison (on the main outcomes) should be done using the Confidence in Network Meta-Analysis (CINeMA) tool (https://cinema.ispm.unibe.ch).

Response 3: We sincerely appreciate the valuable comments. We will determine the overall quality of evidence for each treatment comparison using the Confidence in Network Meta­Analysis (CINeMA) tool (a web application based on the GRADE framework)[7, 8]. (Page 3, lines 44 to 45; Page 17, lines 337 to 398)

Special thanks to you for your valuable comments.

We are very grateful to you and the reviewers for your valuable comments on our paper. If there are any other modifications we could make, we would like to modify them further, and we appreciate your help. We hope that our manuscript can be considered for publication in your journal. Looking forward to hearing from you.

Thank you and best regards.

Yours sincerely,

Honghong Wang

Xiangya School of Nursing, Central South University, 172 Tongzuphill, Yuelu District, Changsha, Hunan Province, P. R. China, 410013.

Email: [honghong_wang@hotmail.com](mailto:honghong_wang@hotmail.com)

**Reference**

1.	Turner RM, Davey J, Clarke MJ, Thompson SG, Higgins JP. Predicting the extent of heterogeneity in meta-analysis, using empirical data from the cochrane database of systematic reviews. Int J Epidemiol. 2012;41(3):818-27. Epub 2012/03/31. doi: 10.1093/ije/dys041. PubMed PMID: 22461129; PubMed Central PMCID: PMCPMC3396310.

2.	Bighelli I, Rodolico A, García-Mieres H, Pitschel-Walz G, Hansen WP, Schneider-Thoma J, et al. Psychosocial and psychological interventions for relapse prevention in schizophrenia: a systematic review and network meta-analysis. Lancet Psychiatry. 2021;8(11):969-80. Epub 2021/10/16. doi: 10.1016/s2215-0366(21)00243-1. PubMed PMID: 34653393.

3.	Dias S, Ades AE, Welton NJ, Jansen JP, Sutton AJ. Network meta-analysis for decision-making: John Wiley & Sons; 2018.

4.	Higgins JPT TJ, Chandler J, Cumpston M, Li T, Page MJ, Welch VA (editors). Cochrane handbook for systematic reviews of interventions version 6.1 (updated September 2020). Cochrane2020. Available from: <www.training.cochrane.org/handbook>.

5.	Wilson DB, Tanner-Smith E, Mavridis D. Network meta-analysis. Campbell Systematic Reviews. 2016;12(1):i-11. doi: <https://doi.org/10.4073/cmpn.2016.1>.

6.	Dias S, Welton NJ, Caldwell DM, Ades AE. Checking consistency in mixed treatment comparison meta-analysis. Stat Med. 2010;29(7-8):932-44. Epub 2010/03/10. doi: 10.1002/sim.3767. PubMed PMID: 20213715.

7.	Puhan MA, Schünemann HJ, Murad MH, Li T, Brignardello-Petersen R, Singh JA, et al. A GRADE working group approach for rating the quality of treatment effect estimates from network meta-analysis. BMJ. 2014;349:g5630. Epub 2014/09/26. doi: 10.1136/bmj.g5630. PubMed PMID: 25252733.

8.	Nikolakopoulou A, Higgins JPT, Papakonstantinou T, Chaimani A, Del Giovane C, Egger M, et al. CINeMA: an approach for assessing confidence in the results of a network meta-analysis. PLoS Med. 2020;17(4):e1003082. Epub 2020/04/04. doi: 10.1371/journal.pmed.1003082. PubMed PMID: 32243458.
